# Retinal and peripapillary vascular deformations in prematurely born children aged 4–12 years with a history of retinopathy of prematurity

**DOI:** 10.1038/s41598-023-30166-1

**Published:** 2023-02-22

**Authors:** Ga-In Lee, Kyung-Ah Park, Sei Yeul Oh, Sang Jin Kim

**Affiliations:** 1 Hangil Eye Hospital, Incheon, Republic of Korea; 2 Department of Ophthalmology, Samsung Medical Center, Sungkyunkwan University School of Medicine, 81 Irwon-ro, Gangnam-gu, Seoul, 06351 Republic of Korea

**Keywords:** Retinopathy of prematurity, Optic nerve diseases

## Abstract

In this study, foveal, parafoveal, peripapillary anatomical, and microvascular anomalies in prematurely born children aged 4–12 years with a history of retinopathy of prematurity (ROP) were evaluated. Seventy-eight eyes of 78 prematurely born children ([tROP]: ROP with laser treatment, [srROP]: spontaneously regressed ROP) and 43 eyes of 43 healthy children were included. Foveal and peripapillary morphological parameters (including ganglion cell and inner plexiform layer (GCIPL) thickness, peripapillary retinal nerve fiber layer (pRNFL) thickness) and vasculature parameters (including foveal avascular zone area, vessel density from superficial retinal capillary plexus (SRCP), deep retinal capillary plexus (DRCP), and radial peripapillary capillary (RPC) segments) were analyzed. Foveal vessel densities in SRCP and DRCP were increased and parafoveal vessel densities in SRCP and RPC segments vessel density were decreased in both ROP groups compared with those of control eyes. The best-corrected visual acuity was negatively correlated with pRNFL thickness in the tROP group. Refractive error was negatively correlated with vessel density of RPC segments in the srROP group. In children born preterm with a history of ROP, it was found that foveal, parafoveal, and peripapillary structural and vascular anomalies and redistribution were accompanied. These retinal vascular and anatomical structure anomalies showed close relationships with visual functions.

## Introduction

Retinopathy of prematurity (ROP) is a hazardous disease that can significantly reduce the quality of life of prematurely born children, despite improvement in survival rate due to medical advances^1^. Several clues of foveal immature appearance have been reported with imaging techniques such as fundus photography and optical coherence tomography (OCT)^2–4^. OCT studies have reported that prematurely born children have thicker central fovea than those born at term regardless of ROP^4,5^. Akerblom et al. have reported that the central macula is significantly thicker in prematurely born children than in full-term children^4^. The thicker central fovea in prematurely born children might be related to halted foveal development referred to as peripheral migration of inner nuclear layer cells at 24 weeks to 26 weeks. Rosen et al. have reported that ganglion cell and inner plexiform layer (GCIPL) thickness is significantly increased in children born extremely preterm^6^. Children with a history of ROP treatment show significantly increased central GCIPL thickness and reduced foveal depths^6^. Recent advances in OCT angiography (OCT-A) to reconstruct three-dimensional vascular structures of retinal layers and peripapillary areas by auto-segmentation have enabled us to measure vessel density quantitatively and reproducibly^7^. A few studies have reported foveal microvascular anomalies in preterm children with^8,9^ or without^10^ ROP using OCT-A. However, Rezar-Dreindl et al. have demonstrated no significant alteration of central vessel density in children born preterm with a history of ROP with or without treatment despite significant alterations in their retinal structures^11^.

The peripapillary retinal nerve fiber layer (pRNFL) thickness is also reduced in prematurely-born children with severe ROP than in children born at term^12^. However, Tong et al. have reported that vertical cup diameter and the cup-to-disc ratio are larger in preterm infants than in term infants after analyzing characteristics of preterm infant optic nerve development measured by OCT^13^. So far, studies focusing on peripapillary vasculatures on OCT-A in prematurely born children with a history of ROP treatment have not been reported yet. Thus, the objective of this study was to evaluate peripapillary, foveal, and parafoveal vascular structures of school-aged children born prematurely who received ROP laser treatment or spontaneously regressed ROP. Relationships of visual function outcomes with vessel density and anatomical structures of peripapillary and parafoveal areas were also evaluated.

## Results

A total of 78 eyes in 78 patients were eligible for inclusion. The distribution of enrolled patients is shown in Table 1. There were no significant differences in the mean examination age or gender between ROP patients and healthy controls. However, there were significant differences in spherical equivalent refractive error (SER) (*p* < 0.0001), best-corrected visual acuity (BCVA) (*p* < 0.0001), and axial length (*p* = 0.0002) at the last visit between ROP patients and healthy controls. SER was the lowest in tROP groups (-3.59 ± 2.73), followed by that in healthy controls (-1.87 ± 3.15) and srROP groups (0.31 ± 1.91). The last BCVA was the lowest in tROP groups (0.25 ± 0.20), followed by that in srROP groups (0.13 ± 0.15) and healthy controls (0.03 ± 0.06). The axial length was the shortest in srROP groups (22.33 ± 1.04 mm), followed by that in tROP (22.57 ± 1.47 mm) and healthy control groups (24.03 ± 1.89 mm). Gestational age (*p* = 0.0105) and birth weight (*p* = 0.0232) were also significantly different between ROP patients and healthy controls.

**Table 1:** Characteristics of preterm patients with a history of retinopathy of prematurity with treatment, spontaneously regressed, and full-term healthy controls.

	tROP patients (n = 38)	srROP patients (n = 40)	Healthy controls (n = 43)	*p* value
Variables	Mean ± SD	Mean ± SD	Mean ± SD	
Gender, male/female	18/20	23/17	24/19	0.6305^a^
Examination age, years	8 ± 2	7 ± 2	8 ± 3	0.1061^b^
Gestational age, weeks	26.00 ± 2.4	26.98 ± 2.2		0.0105^c^
Birth weight, g	867.82 ± 333.5	965.8 ± 228.8		0.0232^c^
Spherical equivalent, diopters	− 3.59 ± 2.73	0.31 ± 1.91	− 1.87 ± 3.15	< 0.0001^b^
Last BCVA, logMAR	0.25 ± 0.20	0.13 ± 0.15	0.03 ± 0.06	< 0.0001^b^
Axial length, mm	22.57 ± 1.47	22.33 ± 1.04	24.06 ± 1.89	0.0002^b^
ROP stage				
Stage 1 (n, %)	0, 0	10, 25		
Stage 2 (n, %)	0, 0	23, 58		
Stage 3 (n, %)	38, 100	7, 18		
Treatment				
Laser	38 (100%)	0		

ROP, retinopathy of prematurity with laser treatment; srROP, spontaneously regressed retinopathy of prematurity; SD, standard deviation; BCVA, best-corrected visual acuity.

^a^ Chi-square test.

^b^ Kruskal–Wallis test.

^c^ Wilcoxon rank sum test.

### pRNFL and GCIPL thickness changes by OCT

Comparisons of pRNFL and GCIPL thicknesses between prematurely born children with ROP treatment or spontaneously regressed ROP and full-term born controls are shown in Table 2. The temporal sector of pRNFL thickness was significantly increased in the tROP group (mean ± SD: 128.68 ± 28.7 μm) than in the srROP group (74.33 ± 18.3 μm; *p* < 0.0001) and the control group (82.42 ± 14.9 μm; *p* < 0.0001). However, it showed no significant difference between srROP and control groups. Superior sector (tROP: 110.68 ± 20.3 μm, HC: 125.67 ± 18.9 μm; *p* = 0.0304) and nasal sector (tROP: 55.87 ± 12.08 μm, HC: 66.58 ± 13.6 μm; *p* = 0.0024) of pRNFL thicknesses were significantly decreased in the tROP group than in the control group. Inferior sector (srROP: 116.75 ± 22.9 μm, HC: 134.95 ± 19.5 μm; *p* = 0.0168) and nasal sector (srROP: 57.98 ± 10.8 μm, HC: 66.58 ± 13.6 μm; *p* = 0.0152) of pRNFL thicknesses were significantly decreased in the srROP group than in the control group.

**Table 2:** Comparison of retinal layer thicknesses in preterm patients with a history of retinopathy of prematurity with treatment, spontaneously regressed, and full-term healthy controls.

Variables	tROP patients (n = 38)	srROP Patients (n = 40)	Healthy controls (n = 43)	tROP vs. HC	srROP vs. HC	tROP vs. srROP	*Adjusted p* value^a^
pRNFL thickness (μm)							
Global	106.69 ± 14.88	91.62 ± 12.76	102.41 ± 10.29	4.14 (2.83)	− 11.26 (2.85)	− 15.40 (2.91)	**< 0.0001**
Superior	110.68 ± 20.3	117.43 ± 21.7	125.67 ± 18.9	**− 15.01 (4.54)** ***p* = 0.0304**	− 8.31 (4.57)	6.70 (4.66)	**0.0052**
Inferior	131.53 ± 34.8	116.75 ± 22.9	134.95 ± 19.5	− 4.06 (5.80)	**− 20.33 (5.83)** ***p* = 0.0168**	− 16.27 (5.95)	**0.0018**
Nasal	55.87 ± 12.08	57.98 ± 10.8	66.58 ± 13.6	**− 11.00 (2.71)** ***p* = 0.0024**	**− 9.58 (2.72)** ***p* = 0.0152**	1.42 (2.78)	**0.0001**
Temporal	128.68 ± 28.7	74.33 ± 18.3	82.42 ± 14.9	**46.65 (4.71)** ***p* < 0.0001**	− 6.82 (4.73)	**− 53.46 (4.83)** ***p* < 0.0001**	**< 0.0001**
GCIPL thickness (μm)							
Global	68.09 ± 21.05	77.54 ± 8.41	83.64 ± 5.34	**− 16.01 (3.56)**	− 6.84 (3.53)	9.17 (3.44)	**0.0001**
Superior	65.39 ± 27.26	78.56 ± 8.45	83.97 ± 6.32	**− 19.11 (4.45)** ***p* = 0.0008**	− 6.26 (4.42)	12.85 (4.31)	**0.0002**
Inferior	59.00 ± 27.23	74.69 ± 12.63	81.90 ± 5.48	**− 23.45 (4.67)** ***p* < 0.0001**	− 8.09 (4.64)	**15.35 (4.52)** ***p* = 0.0248**	**< 0.0001**
Nasal	68.73 ± 21.17	79.69 ± 9.59	85.29 ± 6.14	**− 16.97 (3.68)** ***p* < 0.0001**	− 6.27 (3.65)	10.71 (3.56)	**0.0001**
Temporal	79.20 ± 20.59	77.23 ± 7.01	83.17 ± 5.03	− 4.26 (3.41)	− 6.39 (3.38)	− 2.13 (3.30)	0.1673

tROP, retinopathy of prematurity with laser treatment; srROP, spontaneously regressed retinopathy of prematurity; HC, healthy controls; pRNFL, peripapillary retinal nerve fiber layer; GCIPL, ganglion cell-inner plexiform layer; SE = standard error, CI = confidence interval.

Values are shown as mean ± standard deviation.

^a^*P* value was calculated by multivariate linear regression after adjusting for age and Bonferroni correction multiplied by 3 with 3 subgroups.

In each group comparison, Bonferroni correction was performed by multiplying *p* value by 8 due to multiple testing of outcomes.

Boldface indicates statistical significance.

Otherwise, all sectors except temporal sector of GCIPL thicknesses were significantly reduced in the tROP group than in the control group (superior, tROP: 65.39 ± 27.26 μm, HC: 83.97 ± 6.32 μm, *p* = 0.0008; inferior, tROP: 59.00 ± 27.23 μm, HC: 81.90 ± 5.48 μm, *p* < 0.0001; nasal, tROP: 68.73 ± 21.17 μm, HC: 85.29 ± 6.14 μm, *p* < 0.0001). However, they showed no significant difference between srROP and control groups. In subgroup analysis of tROP and srROP, only the inferior sector of GCIPL thickness in the tROP group was significantly lower than that in the srROP group (tROP: 59.00 ± 27.23 μm, srROP: 74.69 ± 12.63 μm, *p* = 0.0248).

### Vascular parameters measured by OCT-A

FAZ area, average values of foveal and parafoveal SRCP and DRCP vessel densities, and RPC segment vessel density in patients of ROP groups (tROP, srROP) and the control group are shown in Table 3. The FAZ area was significantly reduced in each ROP group (tROP: 0.09 ± 0.07 mm^2^; *p* < 0.0001; srROP: 0.13 ± 0.11 mm^2^; *p* < 0.0001) than in the control group (HC: 0.33 ± 0.11 mm^2^). However, there was no significant difference in FAZ area between tROP and srROP groups.

**Table 3:** Comparison of foveal avascular zone area and vessel densities of the superficial, deep retinal capillary plexus and radial peripapillary capillary segment between preterm patients with a history of retinopathy of prematurity with treatment, spontaneously regressed, and full-term healthy controls.

	tROP patients (n = 38)	srROP Patients (n = 40)	Healthy controls (n = 43)	tROP vs. HC	srROP vs. HC	tROP vs. srROP	*Adjusted p* value^a^
FAZ area (mm^2^)	0.09 ± 0.07	0.13 ± 0.11	0.33 ± 0.11	− 0.24 (0.02) ***p* < 0.0001**	− 0.21 (0.02) ***p* < 0.0001**	0.03 (0.02)	**< 0.0001**
SRCP, % area							
FAZ (foveal)	28.93 ± 5.31	28.72 ± 5.1	21.40 ± 4.16	7.51 (1.09) ***p* < 0.0001**	7.22 (1.09) ***p* < 0.0001**	− 0.29 (1.12)	**< 0.0001**
Parafoveal (average)	46.29 ± 2.07	46.61 ± 2.32	48.97 ± 2.04	− 2.42 (0.48) ***p* < 0.0001**	− 2.70 (0.48) ***p* < 0.0001**	0.27 (0.50)	**< 0.0001**
DRCP, % area							
FAZ (foveal)	23.84 ± 6.02	22.52 ± 6.02	15.86 ± 4.56	6.87 (1.24) ***p* < 0.0001**	8.03 (1.25) ***p* < 0.0001**	− 1.16 (1.28)	**< 0.0001**
Parafoveal (average)	50.79 ± 2.16	49.63 ± 2.50	52.02 ± 2.63	− 1.20 (0.55)	− 2.26 (0.55) ***p* = 0.0002**	− 1.05 (0.56)	**0.0004**
RPC, % area							
Center	30.66 ± 10.54	33.10 ± 11.24	40.79 ± 10.85	− 10.17 (2.52) ***p* = 0.0002**	− 7.99 (2.51) ***p* = 0.0058**	2.18 (2.56)	**0.0002**
Peripapillary (average)	58.53 ± 3.44	57.59 ± 4.19	61.35 ± 2.50	− 2.75 (0.76) ***p* = 0.0012**	− 3.40 (0.75) ***p* < 0.0001**	− 0.65 (0.78)	**< 0.0001**

tROP, retinopathy of prematurity with laser treatment; srROP, spontaneously regressed retinopathy of prematurity; HC, healthy controls; FAZ, foveal avascular zone; SRCP, superficial retinal capillary plexus; DRCP, deep retinal capillary plexus; RPC, radial peripapillary capillary; SE, standard error; CI, confidence interval.

Values are shown as mean ± standard deviation.

^a^*p *value was calculated by multivariate linear regression with adjustment for age and Bonferroni correction multiplied by 3.

Boldface indicates statistical significance.

Vessel density showed similar trend to FAZ area. Foveal vessel densities in SRCP (tROP: 28.93 ± 5.31%, *p* < 0.0001; srROP: 28.72 ± 5.1%, *p* < 0.0001) and DRCP (tROP: 23.84 ± 6.02%, *p* < 0.0001; srROP: 22.52 ± 6.02%, *p* < 0.0001) groups were significantly higher than those in the control group (SRCP: 21.40 ± 4.16%; DRCP: 15.86 ± 4.56%). However, they showed no significant difference between tROP and srROP groups. Regarding the parafoveal area, vessel density (tROP: 46.29 ± 2.07%, *p* < 0.0001; srROP: 46.61 ± 2.32%, *p* < 0.0001) was lower in SRCP than in the control group (48.97 ± 2.04%). Only, DRCP vessel density was significantly reduced in the srROP group than in the control group (srROP: 49.63 ± 2.50%; HC: 52.02 ± 2.63%, *p* = 0.0002) (Table 3).

Average RPC segment vessel densities in center (tROP: 30.66 ± 10.54%, *p* = 0.0002; srROP: 33.10 ± 11.24%, *p* = 0.0058) and peripapillary (tROP: 58.53 ± 3.44%, *p* = 0.0012; srROP: 57.59 ± 4.19%, *p* < 0.0001) areas were significantly reduced in both ROP groups than in the control group (center: 40.79 ± 10.85%; peripapillary: 61.35 ± 2.50%). However, they showed no significant difference between tROP and srROP groups (Table 3).

When compared by areas, parafoveal areas were significantly decreased in superior sector (tROP: 46.78 ± 3.29%, HC: 49.75 ± 2.83%, *p* = 0.0096), nasal sector (tROP: 45.06 ± 3.14%, HC: 47.76 ± 3.22%, *p* = 0.0168), and temporal sector (tROP: 45.28 ± 3.42%, HC: 48.42 ± 3.01%, *p* = 0.0012) of SRCP in the tROP group and temporal sector (srROP: 45.41 ± 3.56%, HC: 48.42 ± 3.01%, *p* = 0.0024) of SRCP in the srROP group than in the control group (Table 4).

**Table 4:** Comparison sectored vessel densities of the superficial, deep retinal capillary plexus and radial peripapillary capillary segment between preterm patients with a history of retinopathy of prematurity with treatment, spontaneously regressed, and full-term healthy controls.

	tROP patients (n = 38)	srROP Patients (n = 40)	Healthy controls (n = 43)	tROP vs. HC	srROP vs. HC	tROP vs. srROP	*Adjusted p* value^a^
SRCP, % area							
Superior	46.78 ± 3.29	47.45 ± 4.18	49.75 ± 2.83	− 2.94 (0.78) ***p* = 0.0096**	− 2.17 (0.78)	0.77 (0.80)	**0.0007**
Inferior	48.04 ± 4.61	47.82 ± 4.72	49.96 ± 4.62	− 1.96 (1.04)	− 2.33 (1.04)	− 0.37 (1.07)	0.0575
Nasal	45.06 ± 3.14	45.75 ± 3.67	47.76 ± 3.22	− 2.72 (0.76) ***p* = 0.0168**	− 2.09 (0.76)	0.63 (0.78)	**0.0011**
Temporal	45.28 ± 3.42	45.41 ± 3.56	48.42 ± 3.01	− 3.17 (0.75) ***p* = 0.0012**	− 3.11 (0.75) ***p* = 0.0024**	0.06 (0.77)	**< 0.0001**
DRCP, % area							
Superior	51.90 ± 4.46	50.56 ± 4.25	53.75 ± 4.13	− 1.78 (0.95)	− 2.88 (0.95)	− 1.09 (0.98)	**0.0109**
Inferior	52.16 ± 4.22	50.99 ± 5.47	53.41 ± 6.13	− 1.24 (1.21)	− 2.40 (1.22)	− 1.16 (1.25)	0.1483
Nasal	48.97 ± 4.25	48.83 ± 4.22	50.22 ± 4.13	− 1.21 (0.94)	− 1.22 (0.95)	− 0.01 (0.97)	0.3296
Temporal	50.12 ± 3.83	48.08 ± 4.34	50.71 ± 4.57	− 0.59 (0.96)	− 2.61 (0.96)	− 2.03 (0.99)	**0.0215**
RPC, % area							
Superior	65.92 ± 6.44	66.58 ± 5.85	70.54 ± 4.28	− 4.50 (1.22) ***p* = 0.012**	− 3.32 (1.21)	1.18 (1.26)	**0.0009**
Inferior	70.30 ± 5.94	66.64 ± 5.96	72.24 ± 4.95	− 1.87 (1.26)	− 5.27 (1.26) ***p* = 0.0024**	− 3.40 (1.31) ***p* = 0.0316**	**0.0003**
Nasal	44.11 ± 6.24	49.22 ± 5.16	51.00 ± 4.27	− 6.90 (1.19) ***p* < 0.0001**	− 1.89 (1.18)	5.02 (1.23) ***p* = 0.0024**	**< 0.0001**
Temporal	53.80 ± 8.77	47.91 ± 6.77	51.63 ± 5.78	2.28 (1.59)	− 3.12 (1.59)	− 5.41 (1.65) ***p* = 0.0492**	**0.0055**

tROP, retinopathy of prematurity with laser treatment; srROP, spontaneously regressed retinopathy of prematurity; HC, healthy controls; FAZ, foveal avascular zone; SRCP, superficial retinal capillary plexus; DRCP, deep retinal capillary plexus; RPC, radial peripapillary capillary; SE, standard error; CI, confidence interval.

Values are shown as mean ± standard deviation.

^a^*p* value was calculated by multivariate linear regression with adjustment for age and Bonferroni correction multiplied by 3.

In each group comparison, Bonferroni correction was performed by multiplying *p *value by 12 due to multiple testing of outcomes.

Boldface indicates statistical significance.

Regarding RPC segment areas, superior sector (tROP: 65.92 ± 6.44%, HC: 70.54 ± 4.28%, *p* = 0.012) and nasal sector (tROP: 44.11 ± 6.24%, HC: 51.00 ± 4.27%, *p* < 0.0001) in the tROP group and inferior sector (srROP: 66.64 ± 5.96%, HC: 72.24 ± 4.95%, *p* = 0.0024) in the srROP group were significantly decreased compared to those in the control group. Comparison between tROP and srROP groups revealed that temporal sector (tROP: 53.80 ± 8.77%, srROP: 47.91 ± 6.77%, *p* = 0.0492) was significantly increased in the tROP group than in the srROP group, whereas nasal sector (tROP: 44.11 ± 6.24%, srROP: 49.22 ± 5.16%, *p* = 0.0024) was significantly decreased in the tROP group than in the srROP group (Table 4).

### Associations of visual function outcomes with vessel density and structural parameters

**Figure 1:**
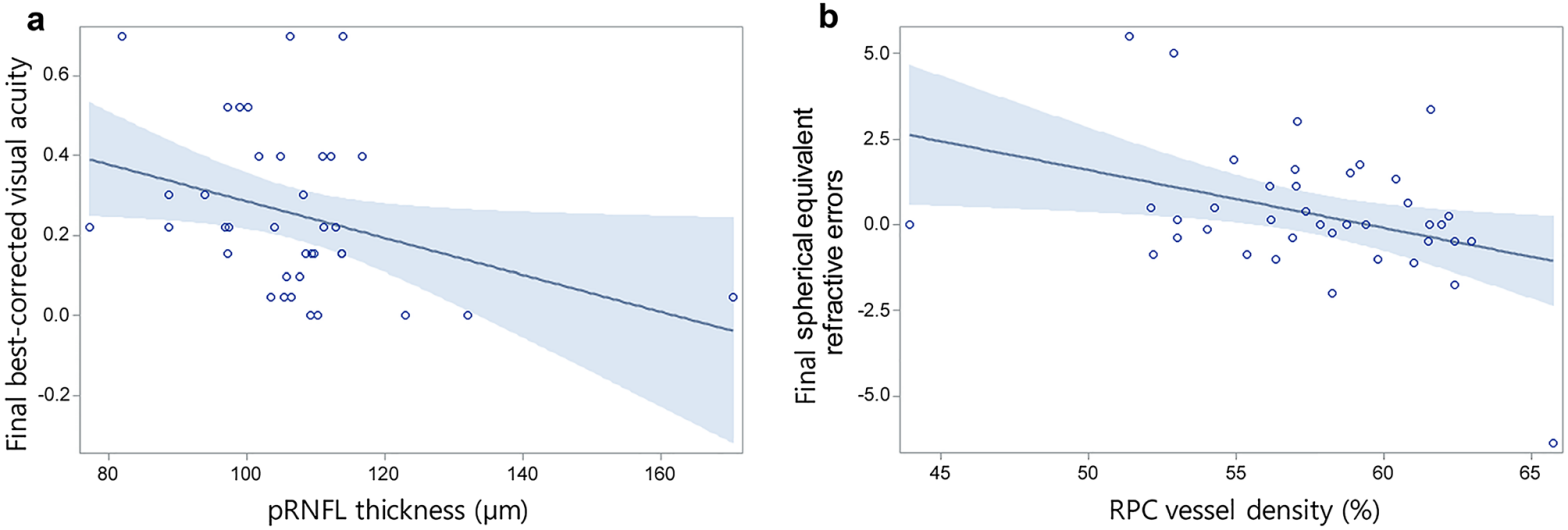
A scatterplot showing associations of visual function outcomes with vascular and structural parameters in retinopathy of prematurity patients. (**a**) Negative association of the peripapillary retinal nerve fiber layer (pRNFL) thickness with final best-corrected visual acuity (*Beta* = − 0.005, *p* = 0.0437) in retinopathy of prematurity (ROP) patients with laser treatment. (**b**) Negative association between radial peripapillary capillary (RPC) vessel density and final spherical equivalent refractive errors (*Beta* = -0.171, *p* = 0.0258) in spontaneously regressed ROP patients. Black line indicates linear regression line. Dark gray area indicates 95% confidence interval.

Relationships of visual function outcomes (including BCVA and SER at the last visit) with vessel density and structural parameters (including pRNFL thickness and GCIPL thickness) were analyzed in both ROP groups. In the tROP group, pRNFL thickness showed a significant negative association with the BCVA at the last visit (Beta, SE: − 0.005, 0.00; *p* = 0.0437). In the srROP group, RPC segment vessel density showed a significant negative association with the SER at the last visit (Beta, SE: − 0.171, 0.07; *p* = 0.0258). Otherwise, visual function outcomes showed no significant associations with foveal or parafoveal vessel densities (Table 5, Fig. 1).

**Table 5:** Association analysis between foveal, parafoveal and peripapillary vessel density and structural, and visual function outcomes in preterm patients with a history of retinopathy of prematurity with or without treatment.

Parameters	*tROP*				*srROP*			
	Final BCVA		Final SER	*p* value	Final BCVA		Final SER	*p* value
	Beta (SE)	*p* value	Beta (SE)		Beta (SE)	*p* value	Beta (SE)	
cSRCP	0.005 (0.01)	0.5255	− 0.003 (0.09)	0.9746	0.002 (0.00)	0.6888	0.019 (0.06)	0.7694
aveSRCP	− 0.019 (0.02)	0.2423	0.085 (0.21)	0.6926	− 0.000 (0.01)	0.9899	0.013 (0.14)	0.9242
cDRCP	0.011 (0.01)	0.0633	− 0.019 (0.08)	0.8214	− 0.001 (0.00)	0.7556	− 0.030 (0.05)	0.5820
aveDRCP	− 0.002 (0.02)	0.9028	− 0.180 (0.20)	0.3783	0.000 (0.01)	0.9845	0.051 (0.13)	0.6857
cRPC	**− 0.007 (0.00)**	**0.0484**	− 0.028 (0.05)	0.5605	− 0.003 (0.00)	0.2801	0.009 (0.03)	0.7654
aveRPC	− 0.004 (0.01)	0.6904	0.106 (0.14)	0.4555	− 0.007 (0.01)	0.2321	**− 0.171 (0.07)**	**0.0258**
pRNFL thickness	**− 0.005 (0.00)**	**0.0437**	0.041 (0.03)	0.1819	− 0.000 (0.00)	0.8601	0.000 (0.03)	0.9891
GCIPL thickness	0.002 (0.00)	0.4657	0.033 (0.02)	0.1943	− 0.006 (0.00)	0.1391	0.012 (0.05)	0.8110

tROP, retinopathy of prematurity with treatment; srROP, spontaneously regressed retinopathy of prematurity; SER, spherical equivalent refractive errors; SE, standard error; BCVA, best corrected visual acuity; pRNFL, peripapillary retinal nerve fiber layer; GCIPL, ganglion cell and inner plexiform layer; SRCP, superficial retinal capillary plexus; DRCP, deep retinal capillary plexus; RPC, radial peripapillary capillary; c, center; ave, average value.

*p* values by linear regression analysis with adjustment for age, birth weight, and gestational age.

Boldface indicates statistical significance.

## Discussion

This study analyzed foveal, parafoveal, and peripapillary vascular parameters in pre- and school-aged children who were born preterm with a history of laser treatment for ROP or with spontaneously regressed ROP and in normal controls who were born at term. In the tROP group, there was a significant increase in temporal pRNFL thickness compared with the srROP or the control group. Other sectors including superior and nasal pRNFL thickness and almost all sectors of GCIPL thickness showed significant decreases in the tROP group than in the control group. In the srROP group, inferior and nasal sectors of pRNFL thickness showed significant decreases compared with those in the control group. Regarding vasculatures, SRCP and DRCP vessel density were significantly increased in the foveal area. However, vessel density was decreased in superficial parafoveal and peripapillary areas of tROP and srROP groups. In the tROP group, thicker pRNFL thickness was associated with better BCVA. In the srROP group, an increase in RPC segment vessel density was associated with a lower SER.

OCT might serve as a tool for screening and assessing structural and functional abnormalities in children with retinal diseases and optic neuropathies. Ecsedy et al. have previously reported results of comparing retinal structures in preterm subgroups and full-term children by OCT^5^. They showed that mean foveal and central retinal thicknesses were decreased in the laser-treated ROP group than in the full-term group^5^. Rosen et al. have demonstrated that extremely preterm children aged 6.5 years receiving ROP treatment show increased GCIPL thickness compared to the extremely preterm group with no or mild ROP^6^. However, the GCIPL thickness was thinner in our patients who received ROP laser treatment compared with control eyes except the temporal sector. The reason for such contradictory results might be related to different patient characteristics between studies such as the proportion of ROP treatment. The previous study included patients with mild ROP as well as severe ROP. Only 13% of patients had received ROP treatment in their study^6^. However, all patients in the tROP group in the present study had severe ROP of stage 3 and received laser treatment. Neuronal damage in this GCIPL area might not only be related to the retinopathy, but also be related to its treatment with diode laser ablation. Pueyo et al. have also reported that children born small for gestational age defined by a birth weight below the 10th percentile without ROP have thinner GCIPL which is correlated with decreased intracranial volumes^14^, suggesting that low birth weight might arrest foveal development and decrease brain volumes.

Mataftsi et al. have reported that there is no significant difference in parafoveal inner-retinal layer thickness among a spontaneously regressed ROP group, a preterm group without ROP, and a normal full-term control group^9^. Another study has also reported that there is no retinal structure difference between preterm infants without a history of ROP treatment and full-term infants^14^. Our study showed similar trends, consistent with previously published studies.

After comparing subgroups between patients with and without ROP treatment, Carreira et al. have reported that the mean macular thickness is higher in the group with laser-requiring ROP than group with non-treated ROP^15^. Our study demonstrated that only inferior quadrant of GCIPL thickness was lower in the tROP group than in the srROP group. Such difference might be affected by the severity of ROP. All patients with tROP had stage 3 ROP. Laser treatment might also play a role in such difference.

Several studies have reported that the development of pRNFL is significantly changed in children born preterm compared to that in children born at term^12,16–19^. Åkerblom et al. have reported that RNFL thicknesses of superior and nasal quadrants are significantly decreased in children with severe ROP than in full-term controls^12^. They suggested that severe ROP with treatment might affect the inner-retinal layer and lead to decreased RNFL thickness^12^. Wang et al. have also reported that in the preterm group, pRNFL thickness is thinner than in full-term controls. However, the temporal sector of pRNFL is 6% thicker than controls, suggesting that preterm birth can disrupt normal neural and vascular development in the eye^19^. Our previous study has demonstrated that the ROP stage is inversely correlated with these abnormalities in RNFL distribution in prematurely born children^16^. Wang et al.^19^ and Park et al.^16^ have reported that the temporal quadrant is significantly thicker in preterm infants with previously treated ROP than in full-term controls. They hypothesized that this was related to inner retinal layers overlaying the fovea referred to as structural redistribution. In the present study, there was also a significant increase in temporal pRNFL thickness in the tROP group compared to that in the srROP group or the full-term control group. Superior and nasal sector pRNFL thicknesses in the tROP group and inferior and nasal sector pRNFL thicknesses in the srROP group also showed significant decreases compared with those in the control group. Between tROP and srROP groups, a significant increase in temporal quadrant pRNFL thickness was found in the tROP group compared with that in the srROP group. In the same manner as the previous hypothesis, abnormalities in the spatial outline of pRNFL might be related to abnormal inner retinal layers maturity. It has been suggested that retinal layers that are immature upon birth might have migrated to the direction of increasing temporal sector of pRNFL. Temporal thickening of pRNFL also indicates immature development of peripapillary neurovascular structures^16,19^.

Recently, several studies have reported alterations of foveal vasculatures using OCT-A in children born preterm with ROP^20–22^. Chen et al. have reported that children born preterm with a history of anti-VEGF agent treatment show significantly higher foveal vessel density than full-term controls^20^. They explained that this tendency appeared to have occurred because of disruption in the balance of VEGF level^20^. Balasubramanian et al. have demonstrated that parafoveal superficial and deep plexus vessel densities between preterm infants with ROP managed by laser treatment and those without ROP or control groups are different only in the foveal area^21^. On the contrary, Nonobe et al. have demonstrated that vessel density does not show any significant difference in ETDRS sector 1 referred to as foveal avascular zone (FAZ) compared to that in controls^22^. However, there were significant decreases in vessel density in ETDRS sectors 2–5 referred as a parafoveal area in children with a history of ROP treatment^22^. The authors suggested that the region with low vessel density was around the macula that was out of FAZ and that it was affected by developmental issues in this region^22^. Similar to previous studies, we found a significant increase in vessel density in superficial and deep foveal areas according to a decrease of FAZ size and a decrease in vessel density in the superficial parafoveal area compared with the control group. However, we found no significant difference in vessel density of superficial or deep foveal area between subgroups of tROP and srROP. It means that it might be hard for laser treatment to affect microvascular structures. These structures are likely due to prematurity itself rather than ROP and/or laser treatment.

One case series has reported foveal development in ROP patients treated with intravitreal bevacizumab or laser photocoagulation^23^. It suggested that immature development of inner retinal layers and delayed development of the ellipsoid zone at the foveal center might have been induced by laser photocoagulation in ROP patients. Lepore et al. have compared 36 eyes of 18 patients with one eye treated with laser photocoagulation and the other eye treated with intravitreal bevacizumab injection for type 1 ROP^24^. Eyes treated with intravitreal bevacizumab tended to have thinner foveal thickness than eyes treated with laser photocoagulation. This result suggests that anti-VEGF therapy might have different effects if there is more foveal development physiologically. However, such differences were minimal between the two groups because of a small cohort.

Results of the present study are in line with above studies, suggesting that retinopathy might alter retinal microperfusion and inner or outer retinal layer development.

Treatment type could be one of the potential factors that affect macular vascularity. Kılıçarslan et al. have reported that there is no significant difference in macular vessel density between treatment groups including laser photocoagulation and intravitreal bevacizumab injection^25^. Anti-VEGF and laser photocoagulation might disrupt physiologic migration of retinal cells and vasculature. However, Zhao et al. have reported that the group with a history of intravitreal ranibizumab has a significantly lower central foveal vessel length density and perfusion density but higher FAZ area than the group with a history of laser photocoagulation^26^. This suggests that intravitreal ranibizumab injection can induce a decrease of VEGF, resulting in decreased foveal vascular density. Since there was no difference according to treatment type in other areas including foveal or parafoveal areas, this central fovea was considered to play an important role in leading to variations of retinal vessel length and perfusion density^26^. However, in this study, we only included ROP patients treated with laser photocoagulation. Thus, it is necessary to study differences in vessel density and neurostructures according to treatment options.

The present study first demonstrated a reduced vasculature of the peripapillary area in prematurely born patients with a history of ROP treatment and a spontaneously regressed ROP. Superior and nasal sectors of peripapillary vascular density were significantly lower in the tROP and inferior sector of peripapillary vessel density was lower in the srROP group than in full-term controls. This trend was similar to pRNFL thickness. It might be partially explained by the fact that a thinner pRNFL in children born preterm is related to subclinical optic nerve hypoplasia^19^. These structural alterations around the optic nerve head can affect vascular development in this area. Due to excessive elimination of axons with altered functional and metabolic requirements, subnormal optic disc and rim areas might be noted in preterm birth^27,28^. A previous cohort study on healthy children aged 7–9 years reported that children with smaller vertical optic disc diameters had narrower retinal arteriolar and venular calibers^29^. Otherwise, redistribution of thicker temporal pRNFL thickness and thinner nasal pRNFL thickness is a vascular parameter reflected by OCT-A. Between subgroups of tROP and srROP, the tROP group was found to have increased temporal sector and decreased nasal sector RPC segment vessel densities than the srROP group.

Peripapillary microvascular alterations in prematurely born patients with ROP in this study might have occurred as a conjunction of disruption in neurovascular development of preterm children. Numerous studies have provided evidence on alterations of structural and functional development in prematurely born patients regarding the visual system^13,16,30–33^ and the nervous system^34–36^. However, whether ROP affected peripapillary perfusion directly or shared background properties in this study was unclear because all subjects in this study had histories of ROP. Further studies including subjects with and without histories of ROP are needed to reveal the true nature of these intraretinal microvascular alterations in preterm patients.

Previous studies have reported that pRNFL thickness has a significant association with visual function^19,37^. Such significant association might be attributed to brain structural and neurodevelopmental changes^31^ as well as irregular macular development in preterm infants^17^. These results suggest that pRNFL alteration can be a potential surrogate marker for visual functional development. In the present study, we also found a significant association between BCVA at the last visit and pRNFL thickness in the tROP group. Regarding the predictive value of foveal OCT-A parameters, Mataftsi et al. have reported that foveal vessel density shows a significant positive correlation with visual acuity in prematurely born children with a history of spontaneously regressed ROP or without ROP^9^. Similarly, Rezar-Dreindl et al. have demonstrated that center foveal superficial and deep vessel densities show significant correlations with visual acuity in prematurely born children with ROP without treatment or with laser treatment^38^.

However, our study found that only peripapillary RPC segment vessel density was associated with refractive error at the last visit. Such results might be explained by the possibility that optic nerve perfusion and structural development are associated with long-term visual outcomes.

This study has several limitations. First, our sample size was relatively small. Regarding parafoveal SRCP vessel density, the only significant difference was found between tROP and healthy control groups. In other groups, parafoveal SRCP vessel density tended to show a difference. However, such difference was not statistically significant. This might be due to the weak statistical power caused by a small number of each group. Second, the age range at which the test was performed was widely distributed due to the retrospective design of this study. Third, vessel density was measured using OCT-A in which the angiographic signal was based on movement. Therefore, we could not exclude false-positive findings due to technical and methodological issues. However, we found that there were peripapillary RPC segment changes in prematurely born patients who received ROP treatment and spontaneously regressed ROP for the first time. Fourth, this study used full-term healthy patients as controls for comparison with two different populations of premature and mature. Therefore, it was challenging to determine whether the outcome feature was due to prematurity itself, ROP feature, or laser treatment. Further study that designs a control group more strictly would be more meaningful to understand the pathophysiology of ROP.

In summary, we found that foveal superficial and deep vessel densities were significantly increased when FAZ size was decreased. We also found that parafoveal and peripapillary vessel densities were significantly decreased in children born preterm with or without ROP treatment compared to those of full-term controls. In addition, we found that peripapillary perfusion was redistributed according to structural change in prematurely born children with a history of ROP compared to children born at term for the first time, suggesting that ROP and treatment might interrupt normal neurovascular development of the area around the optic nerve head as well as the foveal area.

## Methods

This study was approved by the Institutional Review Board of Samsung Medical Center (Seoul, Republic of Korea). Informed consent was waived for patients with ROP by the Institutional Review Board of Samsung Medical Center (IRB No. 2018-07-144-004). This study was conducted in accordance with the tenets of the Declaration of Helsinki. This retrospective cross-sectional study included children 4–12 years of age who were preterm at birth and diagnosed with ROP. They had received laser treatment for ROP or spontaneously regressed. These patients visited the Department of Ophthalmology at Samsung Medical Center from August 1, 2018 to March 31, 2021. Children were included in this study if they had a BCVA of more than 20/200, normal macular aspect on fundoscopy, ability to fixate on the light target, and could maintain a stable head position during image capture. Those with eyes showing abnormal findings in the retina and corneal opacity or cataracts that could affect the media and prevent detailed imaging were excluded. Study subjects with known ocular abnormalities (high myopia and hyperopia as a refractive error greater than 10.0 diopters of spherical equivalent or 3.0 diopters of astigmatism, any retinal disease, glaucoma, or other optic neuropathy) or prior ocular surgery, systemic diseases (such as diabetes, hypertension, cardiovascular disease, and renal disease), or demyelinating diseases were also excluded.

Control eyes were enrolled from healthy volunteers (gestational age ≥ 37 weeks, birth weight ≥ 2500 g) who had visited the clinic for screening ophthalmic examinations. Children aged between 4 and 12 years required parental or guardian informed consent. When appropriate, child assent was obtained before enrollment into the study (IRB no. 2018-04-131-017).

All patients were diagnosed and treated for ROP by a retina specialist (S.J.K). Cyclopentolate 1% and phenylephrine 2.5% were used to dilate eyes before imaging. Cycloplegic refractive errors of eyes were measured (Topcon KR-8800 Autorefractor Keratometer, Tokyo, Japan) and converted to SER as the sum of the sphere value plus half of the cylindrical value. Device calibration was performed daily. At least five measurements were taken for each eye and their mean values were recorded.

Patients with a history of ROP and healthy subjects underwent Cirrus High-Definition (HD)-OCT analysis (Carl Zeiss Meditec AG, Jena, Germany). The OCT instrument was used to scan the macula (macula cube 512 × 128 protocol) and optic disc (optic disc cube 200 × 200 protocol) to obtain macular ganglion cell-inner plexiform layer (GCIPL) and pRNFL measurements. A recognition algorithm was used to detect inner and outer borders of the pRNFL, from a 1.73-mm diameter circle extracted from the optic nerve cube scan centered on the optic nerve head. The distance between the two lines was measured as pRNFL thickness at particular locations around the optic nerve (superior, inferior, temporal, and nasal). Only well-focused, well-centered scans with signal strength above 6 units were used in analyses. The automated macular Ganglion Cell Analysis algorithm distinguished the outer border of the RNFL and the inner plexiform layers (IPL) and calculated the thickness of the ganglion cell layer (GCL) and the IPL. The thickness of the GCIPL was automatically measured at various locations around the fovea (superonasal, superior, superotemporal, inferonasal, inferior, and inferotemporal). Superotemporal and inferotemporal values were averaged and calculated as the value of the temporal area. Superonasal and inferonasal values were averaged and calculated as the value of the nasal area. The average value of the four quadrants was used in the statistical analysis.

**Figure 2:**
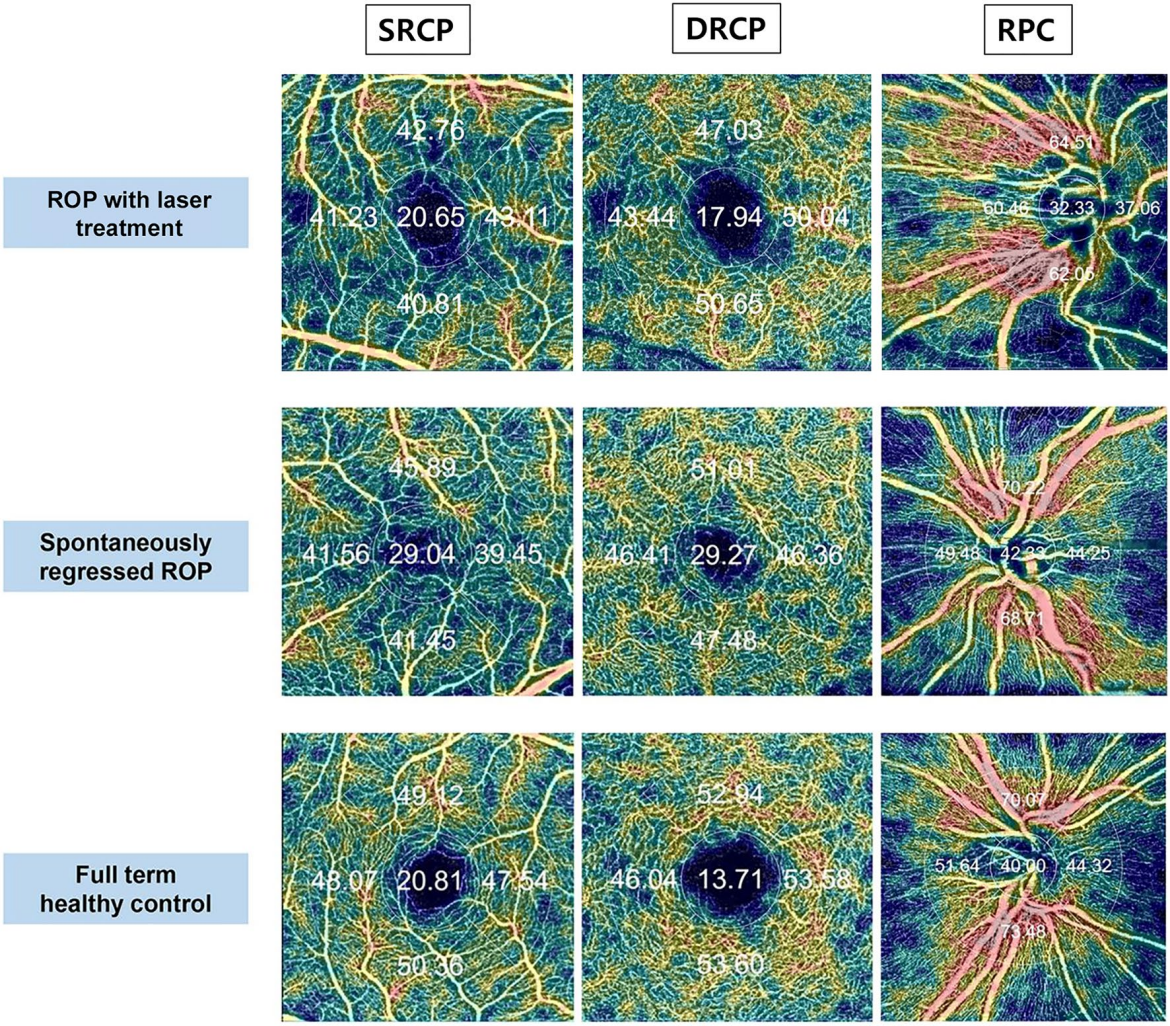
Typical parafoveal and peripapillary en-face example images of an eye with retinopathy of prematurity with or without treatment and a control eye by optical coherence tomography angiography. Color-coded flow maps reveal automated measurement of vessel density with percentage by auto-segmentation including the superficial retinal capillary plexus (SRCP), the deep retinal capillary plexus (DRCP), and the radial peripapillary capillary (RPC) segment.

Swept-source OCT (DRI OCT Triton Plus; Topcon Corporation, Tokyo, Japan) coupled with non-invasive OCT-A technology was used in all children born preterm with ROP and healthy subjects. Details have been described previously^39^. The superficial retinal capillary plexus (SRCP) slab was automatically segmented from 3 µm under the internal limiting membrane (ILM) to 15 µm below the IPL, while the deep retinal capillary plexus (DRCP) slab was automatically segmented from 15 to 70 µm under the IPL in accordance with a method described by Park et al.^40^ The radial peripapillary capillary (RPC) segment ranged from the ILM to the posterior boundary of the RNFL. Vessel density was determined as the percentage of total area occupied by vessels and microvasculature. It is expressed as color-coded vessels quantitatively in a localized region. It was obtained by applying an overlay containing the two inner rings of the Early Treatment Diabetic Retinopathy Study (ETDRS) grid pattern to the fovea automatically, producing a density in each layer with high reproducibility and repeatability^7^. Perifoveal vessel density was determined as the mean of the four sectors in the external ring. The perifoveal ring divided the macular region into superior, inferior, nasal, and temporal sections. The same grid was transferred to the center of the pit in the optic disc (Fig. 2). All participants completed both OCT and OCT-A imaging in one day.

The software generated TopQ image quality values for each OCT-A scan and vessel density measurement (Topcon ImageNet 6; OCT Triton, Topcon Corporation). To assess scan quality, scan images with only one eye per patient were included based on the following quality assessment criteria suggested previously^41^: (1) a fine capillary network was clearly visible and easily distinguished from the background signal (eyes with discontinuities in vessels in images were excluded); (2) the foveal avascular zone and disc margin lied within the central portion of the ETDRS grid applied by the OCT-A software (the location of the foveal avascular zone and optic disc margin were reviewed for accuracy and adjusted manually as needed based on the en face angiogram); (3) the retinal pigment epithelium (RPE) layer of the macular OCT scan was tilted by < 5° to the horizontal plane; (4) less than 10% of the area of the superficial capillary plexus image contained motion artifacts, evident as horizontal white lines or black lines; (5) RPE and individual outer segment layers were clearly visible across the horizontal length of the scan; and (6) TopQ image quality score ≥ 60. Expert graders (G.I.L. and K.A.P.) reviewed and verified all images.

### Statistical analysis

Descriptive statistics are presented as mean and standard deviation (SD). The BCVA was expressed in logarithm of the minimum angle of resolution (log MAR) scale. The Kruskal–Wallis test was used for group comparison of non-normally distributed variables. Otherwise, the Wilcoxon rank sum test was used to compare differences in demographic data (gestational age, birth weight) between ROP patients with and without treatment. Chi-square test was applied to compare categorical variable such as gender. Multivariable linear regression analysis was conducted after adjusting for age to compare inner-retinal layer thickness, FAZ area and vessel densities between the two groups of ROP patients (with and without treatment) and healthy control groups. The 95% confidence interval (CI) was used to determine statistical significance. In addition, corrected *p* values were calculated after Bonferroni’s correction for multiple comparisons of three groups by multiplying uncorrected *p* values. Associations of visual function outcomes with vascular and structural parameters were determined by linear regression analysis. Multivariable analysis was performed for variables with *p* value less than 0.2 in univariable analysis. Statistical significance was considered when *p* value was less than 0.05. All statistical analyses were performed with SAS version 9.4 (SAS Institute Inc, Cary, NC, USA).

### Meeting presentations

American Academy of Ophthalmology, November 13–15, 2020.

## Data Availability

The datasets generated and/or analyzed during the current study are available from the corresponding author upon reasonable request.
